# The Negative Role of Ankyrin-Repeat and SOCS-Box Protein 9 in PAR1 Expression and the MAPK Signaling Pathway in Bovine Granulosa Cells

**DOI:** 10.3390/biology14101344

**Published:** 2025-10-01

**Authors:** Daniela Naranjo Gonzalez, Kalidou Ndiaye

**Affiliations:** Centre de Recherche en Reproduction et Fertilité, CRRF, Département de Biomédecine Vétérinaire, Faculté de Médecine Vétérinaire, Université de Montréal, Saint-Hyacinthe, QC J2S 2M2, Canada; daniela.naranjo@umontreal.ca

**Keywords:** apoptosis, ASB9, cattle, ovary, PAR1, proliferation, signaling pathway

## Abstract

**Simple Summary:**

Ankyrin-repeat and SOCS-box protein 9 (ASB9), a protein involved in regulating biological processes, is induced in bovine granulosa cells by luteinizing hormone (LH). Our study on how ASB9 functions in these cells found that ASB9 induction by LH appears to negatively regulate the expression of protease-activated receptor 1 (PAR1), a protein that normally increases with thrombin treatment. This happens even in the presence of thrombin. Furthermore, research indicates that ASB9 plays a negative role in the MAPK signaling pathway, which is important for cell function. This study also revealed that ASB9 negatively affects the number of granulosa cells by inhibiting their proliferation and increasing their apoptosis (programmed cell death). In summary, ASB9’s function in bovine granulosa cells is to downregulate PAR1, suppress the MAPK pathway, and reduce cell numbers by inhibiting proliferation and promoting apoptosis, suggesting that it plays a significant role in the ovulatory process.

**Abstract:**

Ankyrin-repeat and SOCS-box protein 9 (ASB9) is a member of the ASB family of proteins, which act as a substrate recognition component of E3 ubiquitin ligases and regulate various reproductive processes. ASB9 was previously identified as being induced in bovine granulosa cells (GCs) by LH/hCG, and its binding partners, including protease-activated receptor 1 (PAR1), were reported. The aim of this study was to decipher ASB9’s mechanisms of action in GCs and determine whether ASB9 induction by LH/hCG is necessary for the regulation of PAR1 and the signaling pathways involved in GC function and activity. Cultured GCs were treated with different doses of FSH, LH, and thrombin. RT-qPCR analyses revealed that thrombin increased PAR1 expression, while FSH had no effect on PAR1. Treatment with LH significantly downregulated PAR1, even in the presence of thrombin, possibly via ASB9. The phosphorylation profile of MAPK3/1 in thrombin-treated GCs suggests PAR1-mediated control. ASB9 induction appeared to have a negative effect on the MAPK pathway, although thrombin treatment briefly (within an hour) blocked the negative effect of ASB9 on PAR1. Proliferation assays showed that ASB9 negatively regulated the GC number while increasing apoptosis. These data provide evidence of ASB9’s mode of action and its potent functional effects on PAR1 regulation, GC proliferation, and, potentially, the ovulatory process in bovine species.

## 1. Introduction

In mammals, a surge of luteinizing hormone (LH) is crucial for the final stages of ovarian follicle maturation and ovulation. While baseline levels of both LH and follicle-stimulating hormone (FSH) support the initial growth of follicular cells, it is the significant preovulatory increase in circulating LH that triggers a series of essential events within the follicle [1]. Following the selection of the dominant follicle in a cow during the normal estrous cycle, the ovary releases more estradiol, which raises preovulatory LH levels [2]. This LH surge stimulates cumulus cell expansion, the resumption of meiosis in the oocyte, the eventual rupture of the follicle to release the oocyte, and the differentiation of granulosa cells into luteal cells [3,4,5]. Specific biochemical and molecular alterations in follicular cell function characterize these processes [6]. They involve a complex network of signaling pathways, including protein kinase A (PKA), protein kinase C (PKC), phosphatidylinositol 3-kinase (PI3K), tyrosine kinase-mediated pathways, and mitogen-activated protein kinase (MAPK) signaling pathways, all of which are activated by LH [7,8,9,10].

Consequently, gonadotropin stimulation induces the differentiation of somatic cells in the ovary and regulates the expression of genes that impact follicle development and oocyte maturation [7,9]. For instance, the LH-mediated surge in intracellular signaling causes the rapid downregulation or silencing of genes in GCs that regulate the growth of a bovine dominant follicle, while LH/hCG stimulates an array of genes involved in ovulation and luteinization, as demonstrated in a variety of species, including rodents and bovine species [11,12,13,14,15]. The precise coordination of these events is necessary to produce a mature, fertilizable oocyte and to form the corpus luteum, which is vital for a successful pregnancy [16,17,18]. Therefore, studying gene function during these final stages of follicular development and ovulation is critical to better understanding and regulating ovarian activity.

Data from various laboratories have identified genes that are induced in granulosa cells of bovine ovulatory follicles in response to LH surge or hCG injection, such as ankyrin-repeat and suppressor of cytokine signaling (SOCS)-box protein 9 (ASB9) [11,13,19,20]. ASB9 is a member of the ASB family and is differentially expressed in ovulatory follicles compared to other stages of follicular development [19,20]. The SOCS-box protein superfamily includes 18 protein members that make up the ankyrin-repeat and SOCS-box-containing (ASB) family. These proteins interact with Cul5-RBX2 to form E3 ubiquitin ligases (E3), which play significant roles through ubiquitination-mediated pathways [21,22,23]. However, ASB members interact with a wide range of target substrates, so they have a variety of other functions, including regulation of the cell cycle, carcinogenesis, proliferation, and apoptosis [24,25].

To better understand the role of ASB9 in granulosa cells, we sought to confirm that ASB9 controls granulosa cell function by altering target genes. ASB9 modulates binding partners, such as protease-activated receptor 1 (PAR1), to inhibit GC proliferation. ASB9 also influences MAPK signaling by modulating these partners. PAR1 is a seven-transmembrane-domain G protein-coupled receptor (GPCR) and is uniquely activated by N-terminal proteolytic cleavage [26]. Cleavage of the N-terminus unmasks a tethered ligand that binds to the receptor and triggers intracellular signaling [27,28]. The activation of PAR1 by thrombin has been shown to lead to the activation of the MAPK3/1 signaling pathway, resulting in increased proliferation, while the inhibition of PAR1 and MAPK3/1 activation inhibits cell proliferation and migration [29]. In the ovary, PAR1 and other proteases are critical for the cyclical tissue remodeling that occurs during the menstrual cycle. They are involved in processes such as folliculogenesis, ovulation, and the formation of the corpus luteum [30,31]. PAR1’s activation by proteases like thrombin is thought to be a component of the inflammation-like process of ovulation, promoting the production of chemoattractive and inflammatory factors necessary for follicle rupture [31,32].

Building on our previous finding that ASB9 regulates the activity and function of GCs [20], this study further investigates the role of ASB9 in these cells. We hypothesized that ASB9 influences GC activity by downregulating PAR1, which, in turn, negatively affects the MAPK signaling pathway and limits GC proliferation. Using an in vitro bovine GC model, we analyzed how ASB9 regulates PAR1 and its downstream effects on PAR1-activated signaling pathways, specifically MAPKs, and overall GC function.

## 2. Materials and Methods

### 2.1. Cell Culture

For the in vitro model, bovine ovaries were obtained from a slaughterhouse, irrespective of the stage of the estrous cycle, and granulosa cells (GCs) were collected from medium and large follicles (follicle size ≥ 6 mm, by observation). GCs from six to eight follicles were collected by means of aspiration, pooled, and washed. Cell viability was estimated with 0.4% Trypan blue stain, and cells were seeded at a density of 1 × 10^6^ and cultured in 24-well plates (n = 3 independent experiments) to analyze their gene and protein expression following various treatments.

Serum-free culture. Cultures were performed in serum-free McCoy’s 5A medium supplemented with L-glutamine (2 mM), insulin (10 ng/mL), nonessential amino acids (1 mM), bovine serum albumin (BSA; 0.1%), sodium selenite (4 ng/mL), transferrin (5 µg/mL), androstenedione (100 nM), penicillin (100 IU), and streptomycin (0.1 mg/mL). The cells were incubated at 37 °C in a humidified 5% CO_2_ atmosphere for 6 days. The medium was replaced every 48 h with fresh medium. The treatments consisted of the addition of exact amounts of FSH (1 ng/mL), LH (10 ng/mL), and thrombin (10 mg/mL) in separate experiments and for different durations (15 min, 30 min, 1 h, 6 h, and 12 h), followed by analyses of the target ASB9 and PAR1 proteins and mRNA in the GCs.

Serum-based culture. Cultures were performed in McCoy’s 5A medium (from Thermo Fisher Scientific, Saint-Laurent, QC, Canada) containing L-glutamine (2 mM) supplemented with fetal calf serum (2%), insulin (10 ng/mL), nonessential amino acids (1 mM), sodium selenite (4 ng/mL), transferrin (5 µg/mL), androstenedione (100 nM), penicillin (100 IU), streptomycin (0.1 mg/mL), and follicle-stimulating hormone (FSH; 1 ng/mL). FSH was added to the culture medium to induce LH receptor expression prior to treating the cells with LH. The cells were incubated at 37 °C in a humidified 5% CO_2_ atmosphere for 48 h. The medium was then replaced with serum-free McCoy’s medium without FSH for 18 h, after which LH or thrombin was added without any changes to the medium. Treatments were performed to analyze the LH- and thrombin-induced effects on ASB9 and PAR1 expression in the GCs. The treatments consisted of the addition of exact amounts of LH (10 ng/mL) and thrombin (10 mg/mL) in separate experiments and for different durations (15 min, 30 min, 1 h, 6 h, and 12 h), followed by analyses of the target proteins ASB9 and PAR1, respectively.

### 2.2. RNA Preparation and RT-qPCR Analyses

Total RNA was extracted from in vitro bovine GCs samples using the TRIzol Plus RNA Purification Kit (Invitrogen, Saint-Laurent, QC, Canada) and quantified by determining the absorbance at 260 nm using a NanoDrop spectrophotometer (Thermo Fisher Scientific). Reverse transcription was performed using SMART Scribe Reverse Transcriptase (Takara Bio USA). cDNA levels were analyzed by means of RT-qPCR using SsoAdvanced Universal SYBR Green Supermix (Bio-Rad, Saint-Laurent, QC, Canada) following the manufacturer’s instructions. RT-qPCR data were analyzed using the Livak (2^−ΔΔCt^) method [33] with *RPL19* used as a housekeeping gene [34]. Bovine-specific primer sequences for *ASB9*, *PAR1*, *TNFAIP6*, *AREG*, *RPL19*, *StAR*, *CYP11A1*, and *CYP19A1* were used, as presented in Table 1.

### 2.3. Western Blot Analyses

Granulosa cells from in vitro samples were obtained as described above, and the cells were lysed with M-PER buffer (Thermo Fisher Scientific) supplemented with complete protease inhibitors (Sigma-Aldrich, Oakville, ON, Canada) according to the manufacturer’s protocol. Protein concentrations were determined using a Pierce BCA Protein Assay Kit (Thermo Fisher Scientific). Western blot analyses were performed as previously described [20]. Samples (6 µg of total protein per line) were resolved on Novex WedgeWell 4–20% Tris-glycine mini-gels (Invitrogen) and electrophoretically transferred onto polyvinylidene difluoride membranes (PVDF; Bio-Rad) in a Bio-Rad Wet Blot Transfer Machine. After transfer, the membranes were incubated with blocking buffer (5% nonfat dry milk in TTBS (10 mM Tris-HCL, 150 mM NaCl, and 0.1% Tween-20; pH 7.5)) for 2 h. The membranes were then incubated overnight at 4 °C with specific primary antibodies against bovine ASB9 protein (Abcam; 1 ng/mL; cat. # ab97918), MAPK3/1 and p-MAPK3/1 (both from Cell Signaling; 1:1000; cat. # 9102 and 4695, respectively), and PAR1 (Invitrogen; 0.5 mg/mL; cat. # PIPA5116040). Immunoreactive proteins were visualized by means of incubation with a goat anti-rabbit secondary antibody coupled with HRP (Cell Signaling; 1:1000) for anti-ASB9, anti-MAPK3/1 and anti-p-MAPK3/1, and a rabbit anti-goat IgG (H+L) secondary antibody (Invitrogen; 1:1000) for anti-PAR1 for 2 h at room temperature. Revelation was performed using the SuperSignal West Atto Ultimate Sensitivity Chemiluminescent Substrate (Thermo Fisher Scientific). β-actin was used as a reference protein with anti-β-actin antibodies (ACTB; Santa Cruz, Dallas, TX, USA; 200 µg/mL; 1:2000). Semiquantitative analysis was performed using NIH ImageJ software 13.0.6. The phosphorylation level of MAPK was determined using the phospho-MAPK/total MAPK ratio, presented as a fold change.

### 2.4. Functional Analyses

The pQE system (Qiagen, Montreal, QC, Canada) was used for the cloning of ASB9 and generation of the pQE-ASB9 plasmid construct for ASB9 overexpression in GCs [19]. GCs were collected from ovaries obtained from a slaughterhouse and cultured in 12-well plates at a density of 1 × 10^6^ cells/well in McCoy’s 5A medium as described above (n = 2 independent experiments with triplicate wells for each treatment). The GCs were transfected with the pQE-ASB9 construct using Xfect Transfection Reagent (Takara Bio) according to the manufacturer’s protocol. Nanoparticle complexes from the Xfect transfection kit were applied to the GCs and incubated for 9 h at 37 °C, after which they were removed and replaced with serum-free McCoy’s medium without supplementation for 24 h. Treatments were added without a medium change. The treatments consisted of the addition of exact amounts of LH (10 ng/mL) and thrombin (10 mg/mL) in separate experiments and for different durations (15 min, 30 min, 1 h, 6 h, and 12 h). The cells were then collected for protein extraction and Western blot analyses. The effects of ASB9 overexpression were assessed by analyzing the expression of ASB9 and PAR1. The MAPK pathway was also verified by analyzing the phosphorylation levels of MAPK3/1 (ERK1/2).

### 2.5. Proliferation and Caspase 3/7 Activity Assays

Granulosa cells were plated in 96-well plates (1 × 10^4^ cells per well) in McCoy’s medium and then treated with LH (10 ng/mL) or thrombin (10 mg/mL). Cell numbers were measured using CellTiter Proliferation and Viability Assays (Promega, Madison, WI, USA). Then, 20 μL of MTS assay reagent was added to each well, and the plates were incubated at 37 °C for 2 h. The absorbance at 490 nm was read in a SpectraMax luminometer plate reader (Molecular Devices, San Jose, CA, USA). For caspase 3/7 activity, GCs were also cultured in 96-well plates (1 × 10^4^ cells per well) and treated with LH or thrombin. Caspase 3/7 activity was determined using a luminescent assay (Caspase-Glo 3/7 Assay; Promega Corp.). Caspase-Glo 3/7 reagent (100 μL) was added to the culture plate, and the cells in the culture medium were incubated at room temperature for 1 h before the luminescence of each well was measured at 490 nm in a SpectraMax luminometer plate reader (Molecular Devices).

### 2.6. Statistical Analysis

The statistical analyses for all in vitro experiments were performed using Prism software 10 for macOS (GraphPad Software Inc., CA, USA). Samples or treatments were compared using one-way analysis of variance [35]. When the ANOVA indicated a significant difference [36], the Tukey–Kramer test was used for multiple comparisons of individual means among treatments and times. The data are presented as the means ± SEMs, and variables whose *p* value was <0.05 are marked with an asterisk (*) or with different letters. RT-qPCR data are presented as normalized amounts of the respective genes relative to 2^−ΔΔCt^ using *RPL19* as a housekeeping gene.

## 3. Results

### 3.1. ASB9 and PAR1 Expression Are Differentially Regulated by Gonadotropins (LH and FSH) and Thrombin

We previously performed CRISPR/Cas9-mediated knockdown of ASB9 in GCs to verify the effects of ASB9 silencing on target binding partners [20]. The data showed that ASB9 influences the MAPK3/1 pathway in granulosa cells, presumably by interacting with PAR1 on LH-regulated ovulatory genes. We aimed to investigate the mechanism of action of ASB9 in bovine GCs and its impact on the effects of PAR1 on GC activity. First, we used an in vitro model of GCs collected from follicles. Total RNA was extracted from cultured GCs after FSH, LH, and thrombin treatments (15 min and 1 h). The ASB9 and PAR1 levels in the samples were analyzed by means of quantitative RT-qPCR using the specific primers listed in Table 1.

**Figure 1:**
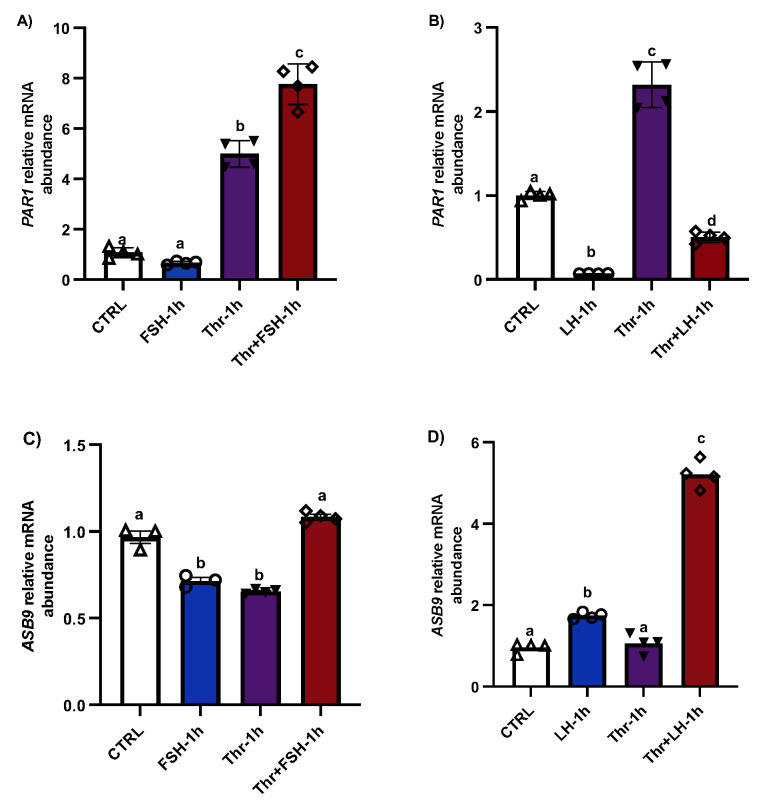
*PAR1* and *ASB9* regulation in granulosa cells (GCs) after FSH, LH, and thrombin treatments. (**A**,**B**) RT-qPCR analyses of in vitro samples indicated greater expression of *PAR1* mRNA in thrombin-treated cells, while treatment with LH significantly decreased *PAR1* expression. *PAR1* mRNA expression increased in cells treated with a combination of FSH and thrombin. (**C**,**D**) RT-qPCR analyses of in vitro samples showed greater expression of *ASB9* mRNA in cells treated with LH than in control cells; however, this expression was greater in the combination of LH with thrombin. *ASB9* mRNA expression decreased in the presence of thrombin or FSH, although the combination of FSH and thrombin resulted in stronger *ASB9* expression than treatment with FSH or thrombin alone. CTRL, control; FSH, follicle-stimulating hormone; LH, luteinizing hormone; Thr, thrombin (n = 4 repetitions for each sample). Different letters (a–d) denote samples that differ significantly.

*PAR1* induction in GCs in vitro was significantly greater following treatment with thrombin for 1 h (Figure 1A, *p* < 0.05), whereas FSH had no effect on *PAR1* expression. However, the combination of FSH and thrombin resulted in a much greater increase in *PAR1* expression than thrombin alone (*p* < 0.05). In contrast, *PAR1* was significantly reduced with LH treatment (1 h), even in the presence of thrombin (Figure 1B, *p* < 0.05), suggesting negative regulation of *PAR1* by LH, possibly via ASB9. The results also showed that *ASB9* expression decreased significantly in the presence of thrombin, while LH induced *ASB9* expression (Figure 1C,D, *p* < 0.05). The combination of FSH and thrombin also increased ASB9 expression, in contrast to the decrease observed when they were applied individually (Figure 1C, *p* < 0.05).

### 3.2. ASB9’s Effects on Target Binding Partners

**Figure 2:**
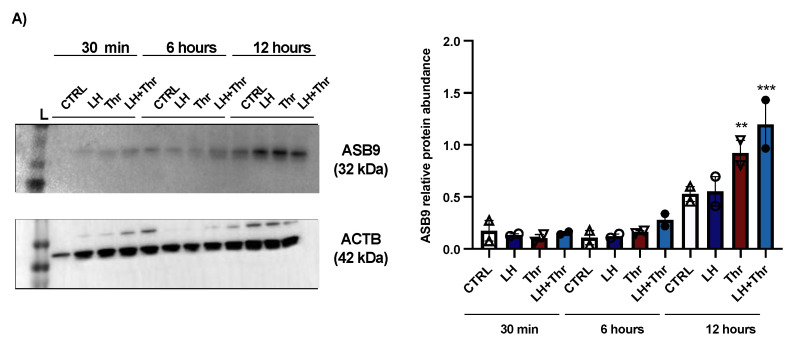

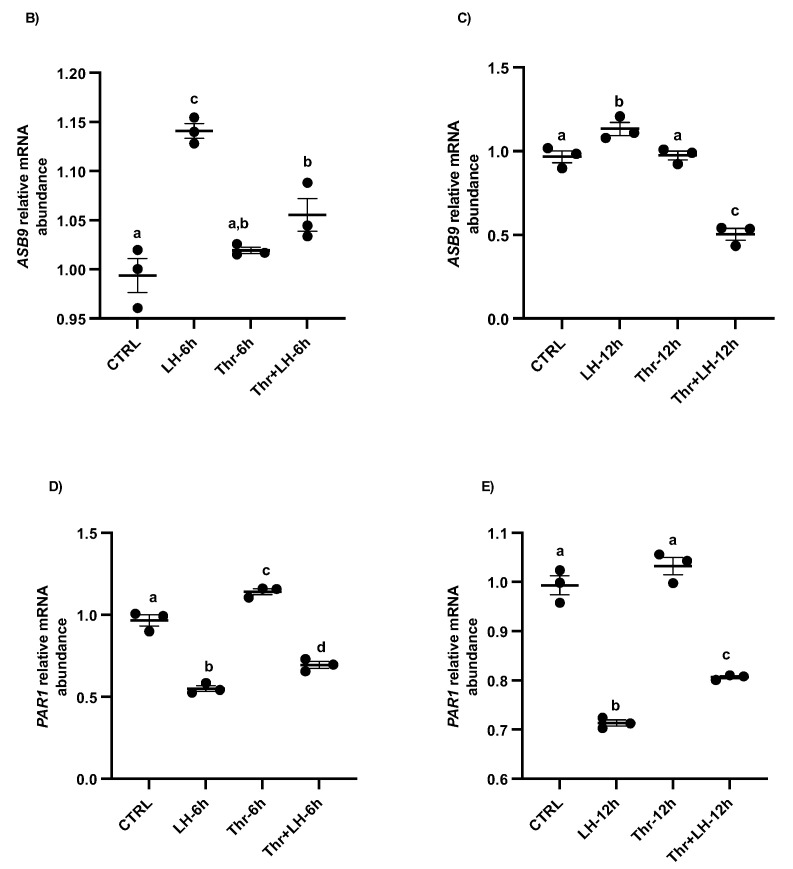
*ASB9* and *PAR1* regulation in granulosa cells (GCs) after LH and thrombin treatments. (**A**) Western blot analyses (Supplementary Materials) of in vitro samples revealed stronger ASB9 induction at 12 h post-LH treatment than at 30 min and 6 h post-LH treatment. The combination of LH and thrombin after 12 h significantly increased ASB9 expression compared to that in the separate treatments. Beta-actin was used as a control. RT-qPCR analyses of in vitro samples showed increased *ASB9* mRNA expression in cells treated with LH at 6 h and 12 h (**B**,**C**). In contrast, thrombin treatment had no effect on *ASB*9 expression, either at 6 h or at 12 h, as compared to the control. Moreover, the results indicated greater expression of *PAR*1 mRNA in cells treated with thrombin, while LH treatment significantly decreased *PAR1* expression at both 6 h and 12 h (**D**,**E**). In vitro samples for RT-qPCR: CTRL, control; LH, luteinizing hormone; Thr, thrombin; ACTB, β-actin; h, hours; n = 3 repetitions for each sample. Different letters denote significant differences among samples; ** significantly different (*p* < 0.001); *** significantly different (*p* < 0.0001).

To better understand the mechanism of action and function of ASB9 in bovine GCs and its effects on PAR1, we performed a second experiment using the same in vitro model of GCs collected from medium and large follicles (follicle size ≥ 6 mm). Total RNA was extracted from the collected GCs after LH and thrombin treatments (15 min, 1 h, 6 h, and 12 h). *ASB9* and *PAR1* expression were analyzed by means of RT-qPCR. In addition to mRNA expression analyses, total protein extracts were analyzed via Western blot using anti-ASB9 antibodies to verify ASB9 regulation in vitro following treatment with LH. The Western blot results revealed that ASB9 protein expression in GCs in vitro increased after 12 h of treatment with LH, as compared to 30 min and 6 h (Figure 2A, *p* < 0.05), confirming previously reported results from our laboratory using an in vivo model [21]. Basal ASB9 expression also increased over time since there was more protein in the control at 12 h than in the control at 30 min. However, the effect of LH induction in combination with thrombin more than doubled the amount of ASB9 protein in the GCs (Figure 2A, *p* < 0.05). On the other hand, the qPCR results indicated greater expression of *PAR1* in cells treated with thrombin, while treatment with LH significantly decreased the expression of PAR1 at 6 h and 12 h, even in the presence of thrombin (Figure 2D,E, *p* < 0.05). This suggests that while thrombin seems to counter the negative effects of ASB9 on *PAR1* early on (1 h), thrombin does not affect ASB9 at 6 h or 12 h when LH-induced ASB9 expression is increased (Figure 2B,C, *p* < 0.05).

### 3.3. Regulation of PAR1 by Thrombin

**Figure 3:**
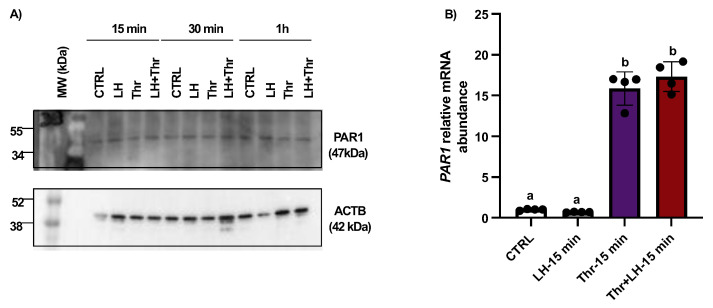
PAR1 protein expression and regulation in bovine granulosa cells post-LH and thrombin treatments. Western blot analyses of in vitro samples showed that PAR1 expression remained unchanged (**A**). However, qPCR data showed that *PAR1* mRNA expression increased significantly in thrombin-treated GCs at 15 min (**B**). In vitro samples: CTRL, control; LH, luteinizing hormone; Thr, thrombin; min, minutes. n = 3 repetitions for each sample. Different letters (a, b) denote samples that differ significantly.

Total protein extracts were analyzed by means of Western blot using anti-PAR1 antibodies. The results revealed no change in the amount of PAR1 at 15 min, 30 min, or 1 h (Figure 3A). Despite the absence of a change in protein expression, thrombin could stimulate PAR1 expression in GCs, as shown by the qPCR analysis, and *PAR1* mRNA expression increased significantly in thrombin-treated GCs at 15 min (Figure 3B).

### 3.4. Thrombin Treatment Increased MAPK3/1 (ERK1/2) Phosphorylation in GCs, Possibly Through PAR1

**Figure 4:**
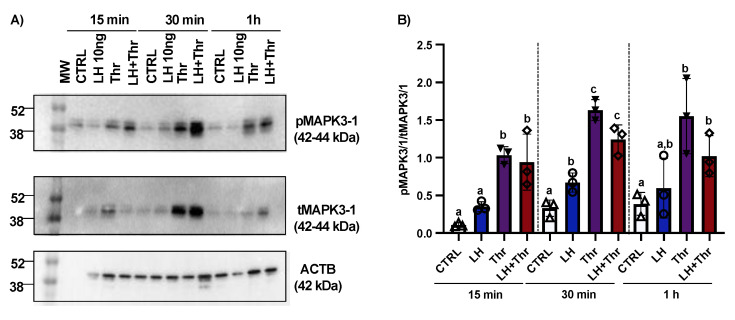
MAPK3/1 protein expression and regulation in bovine granulosa cells post-LH and thrombin treatments. Western blot analyses (Supplementary Materials) using in vitro samples showed an increase in MAPK3/1 phosphorylation in GCs treated with thrombin (**A**,**B**). CTRL, control; LH, luteinizing hormone; Thr, thrombin; ACTB, β-actin; min, minutes; n = 3 repetitions for each sample. Different letters denote significant differences among samples within each time point.

To analyze the regulation of MAPK phosphorylation by PAR1 in GCs, we investigated PAR1 induction using in vitro GC cultures at different times after LH and thrombin treatment (15 min, 30 min, and 1 h; n = 3 for each time point). Total protein extracts were analyzed by means of Western blot using total MAPK3/1 (tMAPK3/1) and phospho-MAPK3/1 (pMAPK3/1) antibodies, and the ratios of pMAPK3/1 to tMAPK3/1 and tMAPK3/1 to β-actin were analyzed and compared among the various treatments and times. Western blot analyses revealed a relative increase in the level of MAPK3/1 phosphorylation in GCs very early following treatment with thrombin (Figure 4A,B). This relative increase in MAPK3/1 phosphorylation was likely modulated by an increase in PAR1 in response to thrombin treatment.

Furthermore, we analyzed MAPK phosphorylation in cultured GCs in response to LH treatment. The analyses revealed that pMAPK3/1 significantly increased at 30 min, while total MAPK3/1 significantly decreased at the same time and at 1 h post-LH treatment (Figure 4). These data are also consistent with previously published data using in vitro samples following treatment with LH at different times (15 min, 30 min, 1 h, and 12 h) in ASB9-inhibited GCs [20].

### 3.5. ASB9 Affects Proliferation, Differentiation, and Apoptosis of GCs In Vitro

**Figure 5:**
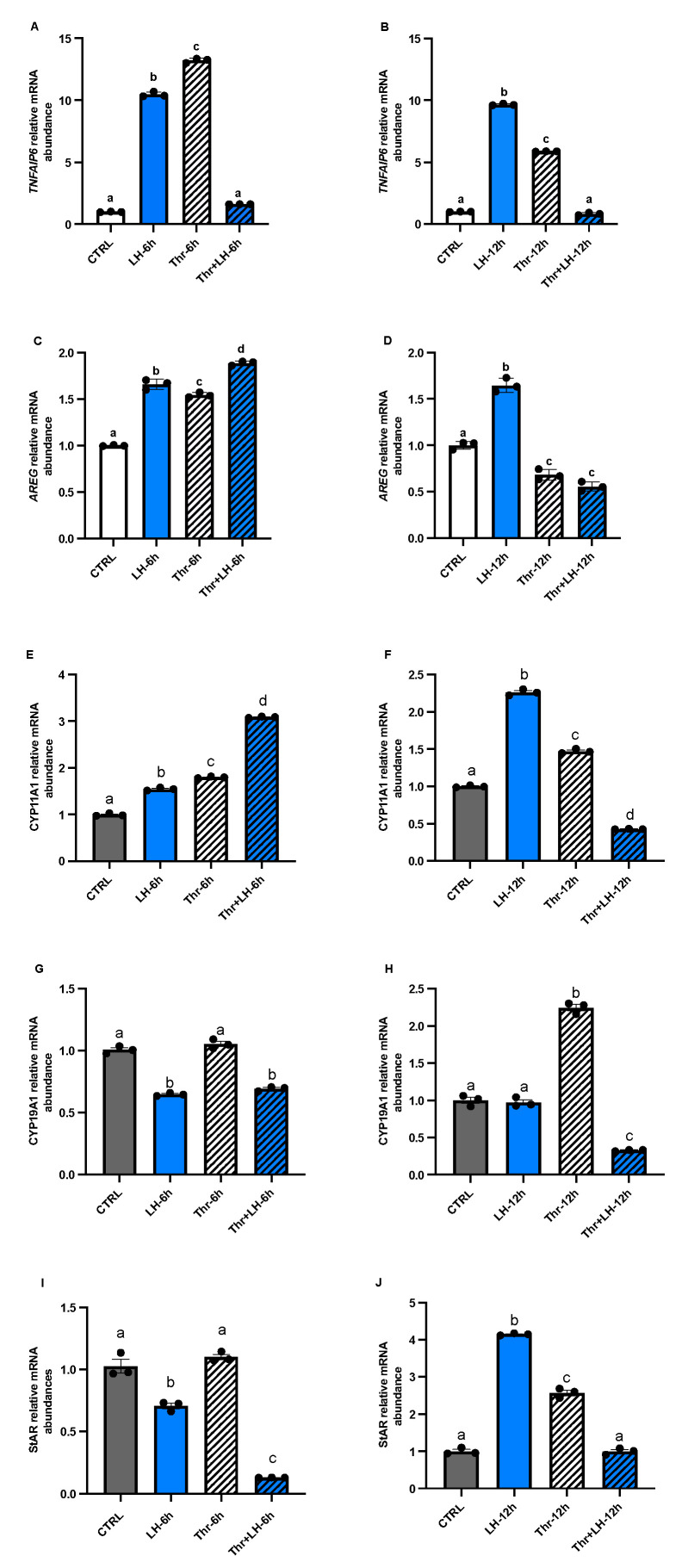
Expression and regulation of the steroidogenesis markers *TNFAIP6* and *AREG*, as well as *CYP11A1*, *CYP19A1*, and *StAR*, in GCs after LH and thrombin treatments. RT-qPCR analyses of in vitro samples revealed that *CYP11A1* mRNA expression (**E**,**F**) increased in the presence of LH or thrombin alone at 6 and 12 h but decreased in the presence of LH combined with thrombin at 12, while *CYP19A1* (**G**,**H**) decreased in the presence of LH at 6 h. *StAR* mRNA expression (**I**,**J**) decreased in cells treated with LH at 6 h but increased in cells treated with LH or thrombin at 1 h. Combined treatment with LH + thrombin reduced *StAR* expression at 6 h but not at 12 h. The results showed greater expression of *TNFAIP*6 (**A**,**B**) and *AREG* (**C**,**D**) mRNAs and LH response genes in cells treated with LH at 6 and 12 h, while thrombin decreased their expression at 12 h. *CYP19A1* mRNA expression was reduced at 6 and 12 h when cells were treated with LH and increased at 6 and 12 h in the presence of thrombin. CTRL, control; LH, luteinizing hormone; Thr, thrombin; n = 3 repetitions for each sample. Different letters denote significant differences among samples.

To analyze the effects of ASB9 on GC activity, we tested the expression of LH response genes *TNFAIP6 (*Figure 5A,B) and *AREG* (Figure 5C,D), as well as steroidogenic enzymes *CYP19A1* (Figure 5E,F), *CYP11A1* (Figure 5G,H), and *StAR* (Figure 5I,J), by means of RT-qPCR. The mRNA levels of *CYP11A1* and *StAR* in GCs in vitro increased after 12 h of LH treatment (Figure 5F,J, *p* < 0.05), while the expression of *CYP19A1* decreased at 6 h (Figure 5G, *p* < 0.05). The expression of *AREG* and *TNFAIP6*, two LH response genes, increased after 6 h and 12 h of LH treatment (Figure 5A–D, *p* < 0.05).

**Figure 6:**
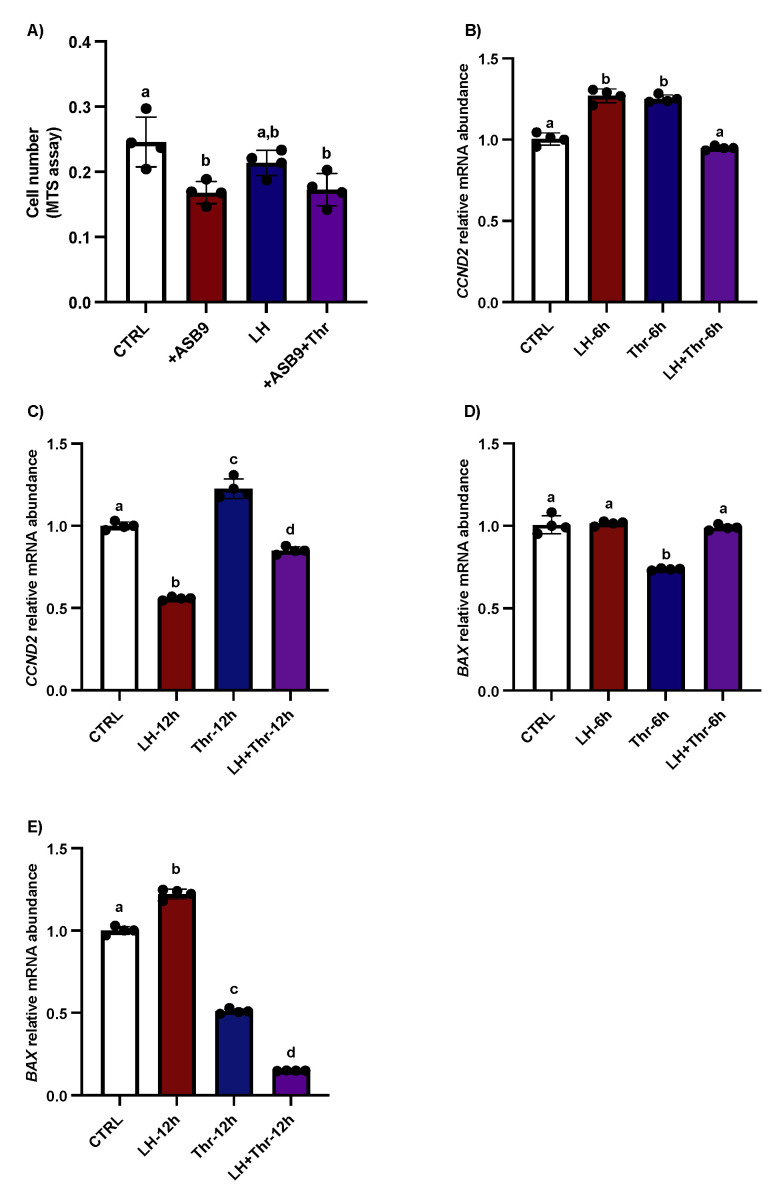
Effects of ASB9 overexpression and LH and thrombin treatments on granulosa cell (GC) proliferation and apoptosis. (**A**) Proliferation assay (MTS assay) analysis showed that the number of GCs decreased following ASB9 overexpression, while the presence of thrombin did not alter the effect of ASB9. (**B**,**C**) RT-qPCR analyses showed that treatment with LH resulted in a significant decrease in *CCND2* mRNA expression after 12 h, while treatment with thrombin for 6 h or 12 h resulted in an increase in *CCND2*. (**D**,**E**) RT-qPCR analyses of in vitro samples showed that *BAX* expression was reduced after thrombin treatment and increased at 12 h in the presence of LH. CTRL, control; LH, luteinizing hormone; Thr, thrombin; +ASB9, ASB9 overexpression; n = 4 repetitions for each sample. Different letters denote significant differences.

We previously carried out yeast two-hybrid screening to identify ASB9’s interactions in granulosa cells [19]. Furthermore, experiments were conducted to analyze the effects of ASB9 on GC proliferation and apoptosis. Proliferation tests showed that following ASB9 overexpression, the number of GCs decreased (Figure 6A, *p* < 0.05); however, ASB9’s activity was unaffected by thrombin. After 6 h, treatment with LH significantly increased the expression of the proliferation marker *CCND2*, whereas at 12 h, *CCND2* expression was reduced (Figure 6B, *p* < 0.05). This result was in contrast with that observed for the thrombin treatment, which increased *CCND2* at both 6 and 12 h (Figure 6C, *p* < 0.05). On the other hand, the effects of LH on ASB9-mediated GC apoptosis were demonstrated, as *BAX* expression increased (Figure 6D, *p* < 0.05) but then decreased at 12 h following thrombin treatment (Figure 6E, *p* < 0.05). However, the caspase 3/7 assay results did not significantly differ among the treatments.

**Figure 7:**
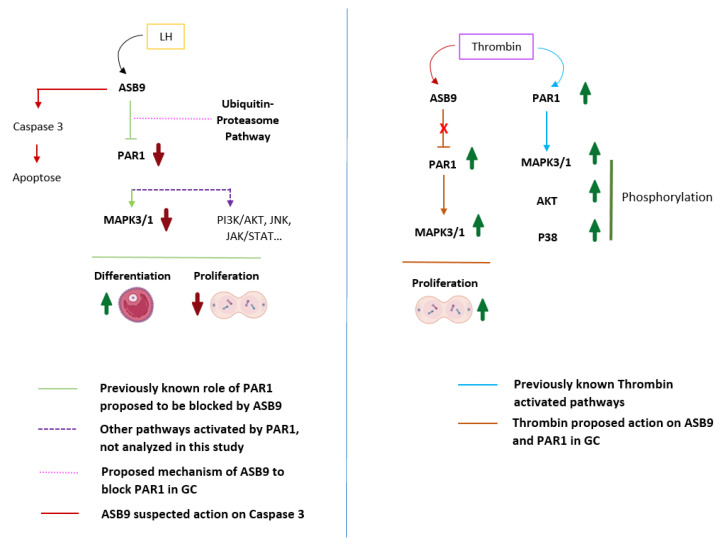
Proposed mechanism of action of ASB9 in GCs on PAR1-induced cell proliferation. PAR1, which couples to G proteins, activates different intracellular signaling pathways, including the MAPK3/1 pathway, leading to cell proliferation. Following induction by LH/hCG, ASB9 binds to and inhibits PAR1, likely through the ubiquitin proteasome pathway of protein degradation, resulting in the inhibition of the PAR1-activated MAPK pathway and the reduction/blockade of granulosa cell proliferation. Granulosa cells expressing ASB9 after LH induction stop proliferating and instead move toward differentiation, which was also evidenced by a decrease in *CCND2* expression. Additionally, we suspect that ASB9 may contribute to cell death by upregulating the expression of proapoptotic markers, as evidenced by the data demonstrating increased *BAX* expression. Thrombin stimulation of PAR1 leads to the activation of different signaling pathways, such as the MAPK3/1, AKT, and P38 pathways. In granulosa cells, thrombin could block the negative action of ASB9, enabling increased proliferation/survival of granulosa cells through PAR1 and MAPK3/1 activation. PAR-1, protease-activated receptor-1; MAPK3/1, mitogen-activated protein kinase 3/1 (ERK1/2); JNK, c-Jun N-terminal kinase; JAK, Janus-activated kinase; STAT, signal transducer and activator of transcription; AKT, protein kinase B; P38, p38 mitogen-activated protein kinase; GC, granulosa cells.

The current data are consistent with previously reported data and support the hypothesis that ASB9 inhibits the MAPK signaling pathway in granulosa cells of ovulatory follicles by targeting specific proteins, such as PAR1, which blocks proliferation and the initiation of GC differentiation (Figure 7).

## 4. Discussion

We previously identified and reported that target genes, including ASB9, are downregulated in GCs from bovine ovulatory follicles following hCG/LH treatment [19,20]. The present study, using an in vitro model, offers new insights into how ASB9 regulates GC proliferation through the PAR1 and MAPK3/1 signaling pathways. We found that ASB9 protein expression in GCs increased after 12 h of LH treatment. These findings mimic and confirm our prior in vivo results, which showed that ASB9 protein expression began to increase in the ovulatory follicle 12 h after hCG injection, continued to rise at 18 h, and reached its strongest induction at 24 h post-hCG [19,20]. These data support the role of ASB9 in regulating the expression of downstream target genes and their signaling pathways in the follicle prior to ovulation. Overexpression of ASB9 (LH-induced gene) led to a decrease in the GC number. LH stimulation led to a decrease in the cell proliferation marker *CCND2* (12 h after LH treatment initiation), an increase in MAPK3/1 phosphorylation (30 min after LH treatment initiation), and an increase in the abundance of the apoptosis marker *BAX* (12 h after LH treatment initiation). These results are in line with previously reported data [20] and demonstrate that, by altering the expression of *CCND2*, ASB9 could have an effect on the G1 phase and the G1-S checkpoint of the cell cycle, and it seems to be associated with a reduction in GC proliferation. Research indicates that CCND2 is essential for ovarian folliculogenesis and plays a significant role in inducing the early-to-mid G1 phase transition [37,38]. Conversely, the increase in *BAX* expression in GCs following ASB9 induction with LH is consistent with the role of ASB9 as a brake on GC proliferation/cell cycle progression to initiate GC differentiation or control GC apoptosis. These observations could also suggest that ASB9 is involved in granulosa cell differentiation into luteal cells; this is similar to its perceived role in mouse spermatogenesis, where overexpression of ASB9 in the spermatogonial stem cell (SSC) line significantly inhibits cell proliferation and increases apoptosis [39]. Additionally, a recent study using porcine GCs showed that ASB9 inhibition increases the GC number by regulating cell cycle-related genes, including *PCNA*, *CCND2*, and *CCNE2* [25].

Since our previous studies identified binding partners for ASB9 in GCs, including PAR1 [13,19,20], we hypothesized that LH-induced ASB9 could regulate GC function by affecting MAPK3/1 signaling through PAR1 to block GC proliferation. PAR1 is a G protein-coupled receptor (GPCR) and is uniquely activated by N-terminal proteolytic cleavage [26]. Cleavage of the N-terminus unmasks a tethered ligand that binds to the receptor and triggers intracellular signaling [40]. The activation of PAR1 by thrombin has been shown to lead to the activation of the MAPK3/1 signaling pathway, resulting in increased proliferation, while the inhibition of PAR1 and MAPK3/1 activation inhibits cell proliferation and migration [29]. According to analyses of preovulatory DNA microarray data, LH/hCG can stimulate the thrombin-THBD-APC-PAR1/4 system in preovulatory follicles [30]. The localization of prothrombin and PAR1 in GCs suggests that these factors may be important mediators of cellular function in the ovarian follicle [41].

In this study, the data showed greater expression of *PAR1* in cultured granulosa cells treated with thrombin, while treatment with LH significantly decreased the expression of *PAR1* at both 6 h and 12 h, even in the presence of thrombin. This suggests that while thrombin seems to counter the negative effects of ASB9 on PAR1 early on (1 h), thrombin does not affect ASB9 at 12 h when LH-induced ASB9 expression is increased. This result supports our previous data from an in vivo model, which showed a reduction in *PAR1* expression in ovulatory follicles 24 h post-hCG compared to day 5 dominant follicles [19]. For a better understanding of PAR1 regulation, we administered thrombin to GCs in addition to LH and FSH. FSH had no effect on *PAR1*, thrombin increased *PAR1* mRNA expression in GCs in vitro, and LH treatment decreased *PAR1*, even in the presence of thrombin, confirming that LH negatively regulates PAR1, potentially through ASB9 induction. ASB9 may have a negative effect on PAR1 through the well-known effect of ASB proteins, which regulate the turnover of a range of proteins by targeting them for polyubiquitination and subsequent proteasomal degradation [42].

Moreover, we analyzed MAPK3/1 phosphorylation in our in vitro model and detected a relative increase in MAPK3/1 phosphorylation in GCs very early following thrombin treatment. This relative increase in MAPK3/1 phosphorylation was likely modulated by an increase in PAR1 in response to thrombin treatment. Additionally, we examined the response of cultured GCs to LH treatment in terms of MAPK phosphorylation. The results of the analyses revealed that at 30 min, pMAPK3/1 significantly increased, while at the same time and at one hour after LH treatment, total MAPK3/1 significantly decreased. These results align with our previously published data using in vitro samples following LH treatment at different times in ASB9-inhibited GCs [20]. The observations from in vitro experiments were comparable to data from GC samples at 24 h post-hCG injection from an in vivo model demonstrating a significant reduction in phospho MAPK3/1 compared to total MAPK3/1 [20]; moreover, ASB9 induction by LH was strongest at 24 h post-hCG [19,20].

LH-dependent phosphorylation and activation of MAPK3/1 have been demonstrated in granulosa cells of different species [10]. Complex transcriptional regulation of target LH-induced genes is required for the control of GC growth and function via various signaling pathways. This regulation influences the optimal physiological status of the follicle before ovulation. The LH-induced MAPK3/1 pathway has been shown to be essential for ovulation [36]. Inhibition of MAPK3/1 in vivo leads to an anovulatory phenotype with trapped oocytes and defective follicular rupture [43]. In mouse preovulatory follicles, LH stimulates the phosphorylation of MAPK3/1 within 30 min, and after two hours of stimulation, the phosphorylation levels increase. MAPK3/1 is activated first in mural granulosa cells and subsequently in cumulus cells over time [10,44]. Similarly, we provide insight into the potential role of PAR1 activation in cell proliferation through the MAPK3/1 signaling pathway. Moreover, it is known from the literature [45,46,47,48] that PAR1 activation by thrombin modulates cell survival through MAPK3/1 signaling pathway activation [49]. Together, these findings suggest that LH-induced ASB9 is involved in regulating granulosa cell proliferation by affecting MAPK3/1 signaling through ASB9 binding partners such as PAR1. Two additional investigations that reported ASB9’s involvement in cancer provided evidence of the detrimental effects of ASB9 on cell proliferation [50,51]. However, other research has demonstrated that thrombin-induced PAR1 activation in astrocytes triggers the MAPK3/1 signaling pathway, which, in turn, promotes the exponential growth of cells [52].

LH triggers the production of ASB9 in bovine granulosa cells, which, in turn, decreases the ability of PAR1 to promote GC proliferation. While PAR1 likely controls the phosphorylation of MAPK3/1 in thrombin-treated GCs, ASB9’s induction by LH reduces this phosphorylation. Thrombin can temporarily counteract ASB9’s negative effects but only for a brief window before ASB9 is fully induced by LH. Overall, the data highlight ASB9 as a crucial regulator of ovarian GC proliferation and activity.

## 5. Conclusions

This research provides strong evidence that ASB9 is a critical regulator of granulosa cell function. Its induction by LH during the preovulatory period is linked to the downregulation of PAR1, a negative effect on the MAPK pathway, and a decrease in GC proliferation while increasing apoptosis. These findings suggest that ASB9 plays a potent functional role in controlling GC activity and may be a key player in the bovine ovulatory process. Future research will investigate the precise mechanisms by which ASB9 regulates PAR1 expression and activity in reproductive cells.

## Figures and Tables

**Table 1:** Primers used for qPCR analysis of Bos taurus gene expression.

Gene Name	Primer Sequences (5′– 3′) ^a^	Accession #	AS (bp)
*ASB9*	Fwd: TCACTGCAGATCGTGTGTCTC; Rv: TCTTAGCAGCTTCGTGGATGG	AY438595	165
*PAR1*	Fwd: GCCTGGCTGACTGTCTTTATC; Rv: AGCACACACACGAAGAGTACG	NM_001103097	170
*AREG*	Fwd: CTTTCGTCTCTGCCATGACCTT; Rv: CGTTCTTCAGCGACACCTTCA	NM_001099092.1	192
*CYP19A1*	Fwd: ATCTGTGCTGATTCCATCACCAAG; Rv: GAAGGAGAGCTTGCCATGCATC	NM_176644.2	167
*CYP11A1*	Fwd: GTGCAAGTGGCCATCTATGCC; Rv: GTGTCCACGTCACCGATATGC	NM_174305.1	161
*TNFAIP6*	Fwd: CTCCAGGCTTCCCAAATGAGT; Rv: GCTGGGTCATCTTCAAGGTCA	NM_001007813	118
*StAR*	Fwd: GGAAAAGACACGGTCATCACT; Rv: AGTTTGGTCCTTGAGGGACTT	NM_174189.3	177
*CCND2*	Fwd: GGGCAAGTTGAAATGGAACCT; Rv: TGGCAAACTTGAAGTCAGTGG	NM_001076372	155
*BAX*	Fwd: TGTCGCCCTTTTCTACTTTGC; Rv: CAAAGATGGTCACTGTCTGCC	NM_173894	200
*RPL19*	Fwd: GACCAATGAAATCGCCAATGC; Rv: ACCTATACCCATATGCCTGCC	NM_001040516	154

Abbreviations: AS, amplicon size (base pairs); Fwd, forward primer; qPCR, quantitative reverse-transcription polymerase chain reaction; Rv, reverse primer; **#**, number. ^a^ All primers were designed and validated by the authors. Each primer was used at a final concentration of 600 nM.

## Data Availability

All the data generated or analyzed during this study are included in this published article.

## Supplementary Materials

The following supporting information can be downloaded at: https://www.mdpi.com/article/10.3390/biology14101344/s1, Western Blot for Figure 2 and Figure 4. Figure S1. Western analysis of ASB9 regulation in granulosa cells (GCs) after treatments with LH and thrombin. Figure S2. Western blot analysis of MAPK3/1 protein expression and regulation in bovine granulosa cells (GCs) after treatments LH and thrombin.

## Abbreviations

The following abbreviations are used in this manuscript:

AREG	Amphiregulin
ASB	Ankyrin-repeat SOCS-box
ASB9	Ankyrin-repeat SOCS-box protein 9
BAX	Bcl-2 like protein 4
CCND2	Cyclin-D 2
CCNE2	Cyclin-E 2
Cul5-RBX2	Cullin-RING ligase 5–RING protein RBX2
CYP11A1	Cytochrome P450 Cholesterol Side-Chain Cleavage, Family 11, Subfamily A, Polypeptide 1
CYP19A1	Cytochrome P450, Family 19, Subfamily A, Polypeptide 1
E3	Ubiquitin ligase
ERK1/2	Extracellular-Signal-Regulated Kinase
FSH	Follicle-stimulating hormone
GC	Granulosa cell
hCG	Human Chorionic Gonadotropin
LH	Luteinizing hormone
MAPK3/1	Mitogen-activated protein kinase 3/1
PAR1	Protease-activated receptor 1
RPL19	Ribosomal Protein L19
StAR	Steroidogenic Acute Regulatory protein
SOCS	Suppressor of cytokine signaling
TNFAIP6	Tumor Necrosis Factor-Inducible Gene 6 Protein
