# Supplementary figures and images for: The Negative Role of Ankyrin-Repeat and SOCS-Box Protein 9 in PAR1 Expression and the MAPK Signaling Pathway in Bovine Granulosa Cells
Source: Biology (Basel). 2025 Oct 1;14(10):1344. doi: 10.3390/biology14101344 (PMC12561142; doi:10.3390/biology14101344)

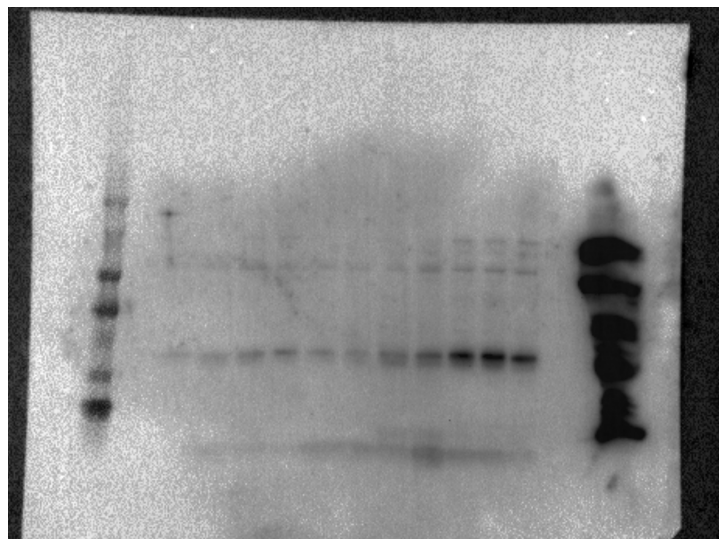

Supplement: Supplementary file 1 [file biology-14-01344-s001.zip › Figure S1.pdf]

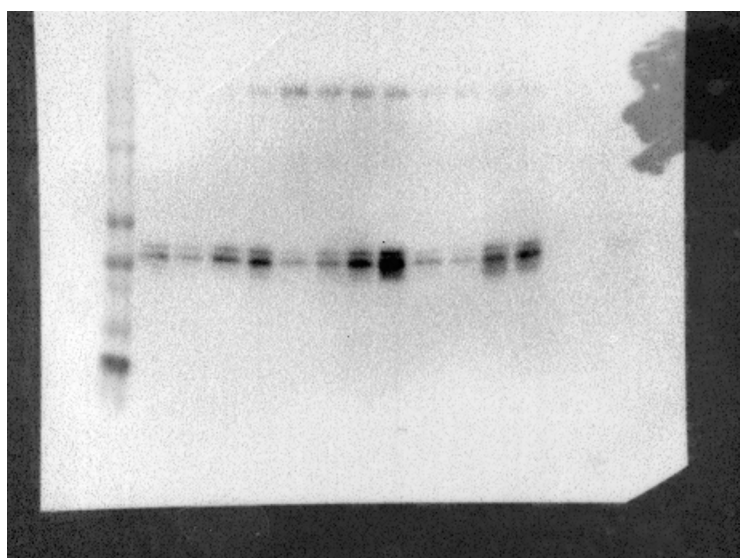

Supplement: Supplementary file 1 [file biology-14-01344-s001.zip › Figure S2.pdf]
